# A Case of Spontaneous Perinephric Hematoma From Rivaroxaban

**DOI:** 10.7759/cureus.29429

**Published:** 2022-09-21

**Authors:** Pei Shan Lee, Shashidhar Baikunje, Swee Ping Teh, Thomas Chan

**Affiliations:** 1 Renal Medicine, Sengkang General Hospital, Singapore, SGP; 2 Urology, Sengkang General Hospital, Singapore, SGP

**Keywords:** direct oral anti-coagulants, acute kidney injury, renal angiography and embolization, rivaroxaban, spontaneous perinephric haematoma

## Abstract

Spontaneous perinephric hematoma is an uncommon but serious condition that is typically caused by tumor hemorrhage or vascular disease such as polyarteritis nodosa. We describe a 78-year-old Chinese gentleman with underlying chronic kidney disease, renal cysts, non-obstructive renal stones, hypertension, ischemic heart disease, and atrial fibrillation who was on rivaroxaban and clopidogrel. He developed spontaneous perinephric hematoma complicated by acute kidney injury, anemia, and myocardial infarction who underwent angioembolization and packed cell transfusion. He did not have further bleeding episodes, and his kidney function improved before discharge.

## Introduction

Spontaneous subcapsular or perinephric hematoma (SPH) in a native kidney is an uncommon but potentially serious clinical condition. Common causes include an underlying renal tumor (e.g., angiomyolipoma or renal cell carcinoma), renal vascular disease (e.g., arteritis), and trauma [1]. Unlike warfarin, currently, there are limited reports on the risks of SPH due to direct oral anticoagulation (DOAC) in native kidneys [2-4]. 

We report our experience of managing a patient on rivaroxaban, a type of DOAC, and clopidogrel who developed a spontaneous perinephric hematoma and was treated with angioembolization successfully.

## Case presentation

A 78-year-old Chinese man presented with acute, localized left-sided lower abdominal pain associated with nausea. There was no history of trauma, fever, hematuria, change in bowel habits, or rectal bleeding. On examination, there was left iliac fossa tenderness and a palpable mass. There was no other organomegaly. He was hypotensive with the lowest recorded blood pressure of 93/57 and tachycardic.

His comorbidities included hypertension, chronic kidney disease, and ischemic heart disease with reduced ejection fraction, for which he had undergone percutaneous coronary intervention with stent insertion and atrial fibrillation. He also has benign prostate hypertrophy (BPH) and bladder stones for which he had undergone cystolithopaxy in 2006. His chronic medications included rivaroxaban, clopidogrel, furosemide, hydralazine, bisoprolol, atorvastatin, empagliflozin, Duodart (dutasteride and tamsulosin), and omeprazole. The patient has been on rivaroxaban 20 mg once a day for six years for atrial fibrillation. He was started on aspirin 100 mg once a day and clopidogrel 75 mg once a day after a myocardial infarction eight months prior. Aspirin was stopped after three months and clopidogrel continued. Follow-up appointments revealed stable hemoglobin levels prior to the admission.

Biochemical investigation as shown in Table 1 revealed low hemoglobin levels, normal platelet levels, and normal activated partial thromboplastin time (aPTT) but increased prothrombin time (PT) and international normalized ratio (INR). His creatinine on admission was raised but at his baseline and consistent with his known history of chronic kidney disease. He also had metabolic acidosis attributed to lactic acidosis from hypoperfusion. 

**Table 1:** Results of laboratory investigations with reference ranges. CKD-EPI: chronic kidney disease epidemiology collaboration.

Laboratory investigation	Results on day of admission	Results on day 2 (day of angiogram and embolization)	Results on day 3 (day after angiogram and embolization)	Results on day of discharge	Reference range
Creatinine (µmol/L)	129	190	302	140	59–104
CKD-EPI estimated glomerular filtration rate (eGFR) (ml/min/1.73 m^2^)	46	29	16	41	-
Bicarbonate (mmol/L)	19.2	17.2	17.6	23	19–29
Lactate (mmol/L)	4.5	6.6	1.0	-	0.5–2.2
Hemoglobin (g/dL)	10.9	7.0	7.8	10.8	14–18
Prothrombin time (PT) (s)	16.6	-	12.4	11.4	9.9–11.4
Activated partial thromboplastin time (aPTT) (s)	32.6	-	31.3	29.2	25.7–32.9
International normalized ratio (INR)	1.63	-	1.19	1.09	-
Platelet (x10^9 ^L)	160	171	117	214	140–440

**Figure 1:**
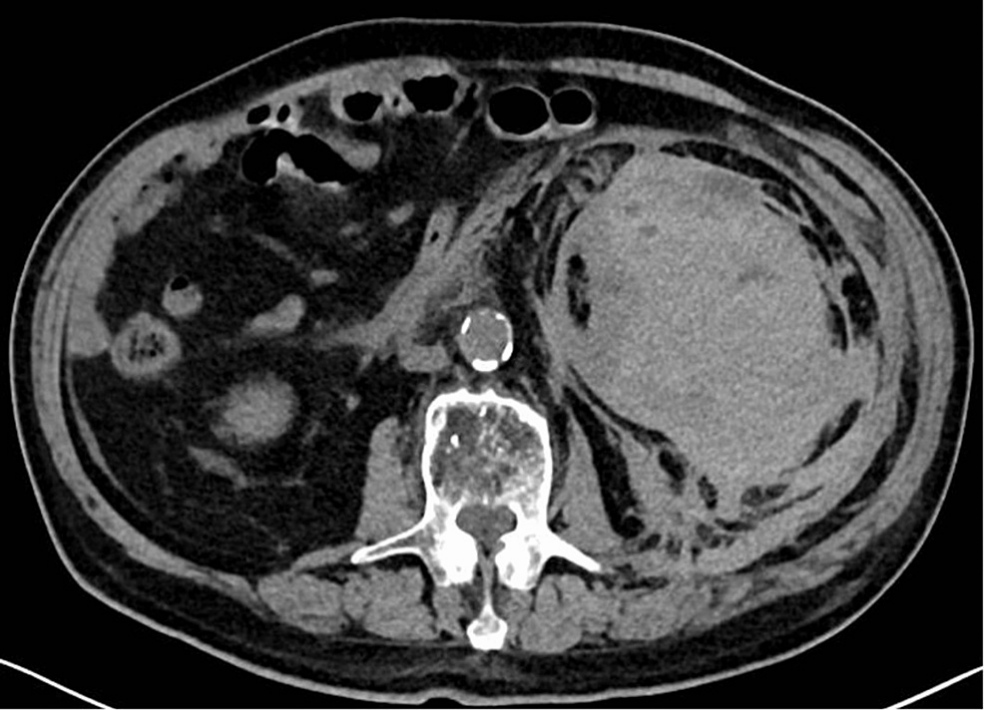
CT image of left perinephric hematoma. CT: computed tomography.

A computed tomography (CT) mesenteric angiogram showed a large perinephric hematoma measuring 13.0 cm × 8.0 cm × 13.6 cm with multiple sites of contrast extravasation with evidence of contrast pooling indicative of active bleeding (Figure 1). There was an extension of the bleed into both the anterior and posterior renal fasciae. A dual left renal artery was noted, but there was no obvious aneurysm seen. There was a reduced enhancement of the left kidney relative to the right. There were also small bilateral small renal cysts noted and several small non-obstructing left renal calculi.

Rivaroxaban and clopidogrel were stopped. He was also given a packed red cell transfusion, a four-factor prothrombin complex concentrate (PCC), and a platelet concentrate transfusion.

He underwent urgent left renal angiography via the right common femoral artery and had a successful embolization of the left main and accessory upper pole of the renal arteries with polyvinyl alcohol (PVA) particles and microcoil. A post-procedure angiogram showed no further bleeding. There were no angiographic features suggestive of arteritis.

During his admission, he developed non-oliguric acute kidney injury (AKI) (Table 1) and myocardial infarction, attributed to hemodynamic instability from acute blood loss, both of which were treated conservatively. He received nine units of packed red blood cells in total. His AKI improved (Table 1) and he did not have further bleeding episodes after angioembolization. Rivaroxaban and clopidogrel continued to be held off on discharge. 

He was subsequently discharged, stable with urology follow-up and continued to be asymptomatic.

## Discussion

Spontaneous perinephric hematoma (SPH) typically presents with Lenk’s triad of flank pain, tenderness, and symptoms of hemorrhage, although there have been various presentations described.

The common causes of SPH include tumor hemorrhage (benign and malignant), vascular disease, infection, and more rarely, noted in patients with renal cystic disease, systemic lupus erythematous, or patients on hemodialysis [1]. 

Diagnosis is usually established radiologically. Ultrasound is useful for quick identification, but CT is more sensitive and can give information about the underlying etiology. Magnetic resonance imaging (MRI) can be a useful alternative to CT. Angiography can be considered should CT fail to localize the underlying cause. Other options include surgical exploration.

DOACs are known to increase the risk of spontaneous hemorrhage, including intra-abdominal hemorrhage [2], but there is limited literature of DOACs causing SPH specifically. Saxena et al. describe a case of SPH in the transplanted kidney due to dabigatran, a direct thrombin inhibitor, which is also a DOAC [3]. Chenna et al. described a similar case of an elderly gentleman with underlying chronic kidney disease on rivaroxaban for atrial fibrillation with SPH who was successfully managed with supportive care [5]. Iplikci et al. described a patient with diabetes and hypertension, who started on edoxaban for atrial fibrillation, who developed SPH and subsequently demised from heart failure and pneumonia despite the team’s best efforts [4]. 

Clopidogrel reduces adenosine diphosphate (ADP)-induced activation of the membrane Gp IIb/IIIa complex leading to irreversible inhibition of ADP-induced binding of platelets to fibrinogen. Ibrahim et al. describe a series of cases with spontaneous perirenal hematoma secondary to anti-platelet therapy, which includes aspirin, clopidogrel, or both. In this literature review, two of the reported cases were on clopidogrel only for four years and six years prior to their presentation with spontaneous perirenal hematoma [6]. 

Our patient had risk factors of being on both rivaroxaban and clopidogrel, hypertension, and renal cystic disease. He did not have features suggestive of infection, tumors, or vascular disease noted in the radiological studies, nor did he have any history of bleeding. This suggests that rivaroxaban, especially used concurrently with clopidogrel, may be the most likely cause of his SPH.

Caution should be exercised in using DOACs such as rivaroxaban in patients with underlying renal disease, especially if it is used concurrently with anti-platelets such as clopidogrel and aspirin. Angioembolization, if the source of bleeding can be localized, can be a potential therapeutic option. Nephrectomy may be indicated if angioembolization is unable to stop the bleeding.

## Conclusions

Early diagnosis of spontaneous perinephric hematoma requires detailed clinical evaluation and the use of appropriate radiological studies. One should have a high index of suspicion for SPH in a patient with the relevant risk factors presenting with abdominal pain. In appropriate cases, angioembolization can be life-saving. Further studies should be done to assess if DOACs increase the risk of SPH. 
